# Papillary Tumor of the Pineal Region Identified by DNA Methylation Leads to the Incidental Finding of Germline Mutation *PTEN G132D* Associated with PTEN Hamartoma Tumor Syndrome: A Case Report and Systematic Review

**DOI:** 10.3390/curroncol32030172

**Published:** 2025-03-17

**Authors:** Nikole O’Neal, Eric Goold, Fatemeh Zarei Haji Abadi, Jeffrey Okojie, Jared Barrott

**Affiliations:** 1 SW Idaho Biomedical & Biosafety Center, Twin Falls, ID 83301, USA; 2 Biomedical and Pharmaceutical Sciences, College of Pharmacy, Idaho State University, Pocatello, ID 83209, USA; 3 Department of Pathology, University of Utah and ARUP Laboratories, Salt Lake City, UT 84108, USA; eric.goold@hsc.utah.edu; 4 Department of Molecular Pharmaceutics, College of Pharmacy, University of Utah, Salt Lake City, UT 84112, USA; u1497218@utah.edu; 5 Department of Cell Biology and Physiology, College of Life Sciences, Brigham Young University, Provo, UT 84602, USA; jokojie@byu.edu; 6 Simmons Center for Cancer Research, Brigham Young University, Provo, UT 84602, USA

**Keywords:** papillary tumor of the pineal region, neuropathology, PTEN hamartoma tumor syndrome, molecular analysis, DNA methylation profiling, next generation sequencing

## Abstract

Distinct subgroups of rare brain tumors can be molecularly classified using whole genome DNA methylation profiling and next-generation sequencing. Furthermore, these tools can identify germline mutations contributing to carcinogenesis. Access to molecular testing in the clinical setting is vital for pathology laboratories to make an accurate diagnosis. One molecularly unique brain tumor requiring such tools is the papillary tumor of the pineal region (PTPR). Herein, we present a case report of a 21-year-old male presenting with macrocephaly and obstructive hydrocephalus due to the PTPR. Next-generation sequencing identified a pathogenic *PTEN* p.G132D mutation in the tumor and matched germline findings further identified *PTEN* Hamartoma Tumor Syndrome (PHTS). The case report tumor was initially misdiagnosed as ependymoma while methylation profiling classified it more specifically as a PTPR, Group B. To better understand the current status of PTPRs, we conducted a systematic review of recent cases reporting on the diagnostics, treatments, and outcomes for PTPR patients. To our knowledge, this is the first case report for PTPRs revealing an association with PHTS. Our review revealed inconsistencies in diagnostics, treatments, and outcomes for PTPR, and an underutilization of definitive molecular testing.

## 1. Introduction

An accurate diagnosis is critical for the care and management of patients with intracranial brain tumors. There are both similarities and variabilities in histopathology for many central nervous system tumors, offering added challenges in diagnoses. 

In 2021, the World Health Organization (WHO) listed certain disease entities in the WHO Classification of Tumors: Central Nervous System Tumors 5th edition (CNS5) with genetic and epigenetic characteristics listed as essential and desirable diagnostic criteria for many primary CNS tumors [1]. With over 100 unique primary CNS tumors, ongoing research continues to reveal distinct classifications and subtypes via molecular analysis, which has improved the accuracy of clinical diagnosis and the survival of patients through improved diagnostic accuracy and targeted therapies [1,2].

One primary CNS tumor described in 2016 as a “distinct molecular entity” is the papillary tumor of the pineal region (PTPR) [3]. The PTPR is a rare neuroepithelial tumor that most commonly arises in the posterior aspect of the third ventricle near or around the pineal region [4]. It is often seen obstructing the cerebral aqueduct, requiring immediate surgical intervention to reduce intracranial pressure from obstructive hydrocephalus [4,5,6,7].

Relying solely on histological analysis, a PTPR can be misdiagnosed. While the WHO has yet to establish a molecular profile for PTPR diagnosis, there is consensus that the loss of chromosome 10 can distinguish these neoplasms [8,9,10]. The aims of this report are to present the background and diagnostic details of a unique and diagnostically comprehensive clinical case. Furthermore, a systematic review on the diagnostics, treatment, and patient outcomes in a five-year timeframe for PTPR is provided.

## 2. Background on PTPRs and PHTS

The PTPR was discovered in 2003 and formally classified in 2007 in the fourth edition of the WHO Classification of Tumors of the CNS [11]. The papillary-like morphology and perivascular pseudorosette patterns appeared distinct with incidence rates slowly increasing since the tumor has been codified [12,13].

The PTPR is currently graded 2 or 3 under the current classification in the WHO CNS5, though this is still not fully defined, with many questions remaining unanswered concerning the behavior of PTPRs [1,14]. Histopathology reports from early published cases indicate PTPRs featuring pleomorphic morphology with distinct perivascular pseudorosettes, variable presentation for necrosis, and moderate to high mitotic activity [5,12,13,15]. Immunohistochemistry (IHC) can appear microscopically similar to ependymomas, suggesting the PTPR likely comes from an ependymal lineage [5,13,16]. It has been suggested repeatedly that specialized ependymal cells associated with the subcommissural organ could be the cell of origin, though this remains largely unknown [5,13,16,17].

PTPR patients require surgical and post-surgical management with variability reported in diagnostics, treatments, and outcomes [5,7]. Unfortunately, evidence is limited for treatments, but various surgical interventions are routinely deployed due to the emergence of secondary hydrocephalus. Overall, gross total resection (GTR) is the recommended treatment for PTPRs in addition to endoscopic third ventriculostomy (ETV) that permanently alleviates pressure build-up from obstruction in the aqueduct [18,19].

With the advent of DNA methylation profiling methods to identify the wide variety of distinct methylation classes, researchers continue to characterize the key attributes of PTPRs. Just recently, one group successfully profiled 76 PTPR tumors to further characterize the subgroup B for PTPRs as B1 and B2 and further revealed the heterogeneity and variability of PTPRs [10].

Morphological similarities to other tumor types of an ependymal lineage create challenges for an accurate PTPR diagnosis via histology alone. As such, we can anticipate that WGMP, as a clinically validated laboratory developed test, will become a critical diagnostic tool in the differential diagnosis of PTPRs in the future.

Due to the incidental findings in this case, we must also discuss the background of *PTEN* hamartoma tumor syndrome (PHTS). *PTEN* is a highly regulated tumor suppressor gene with a wide range of functions and is the second most mutated tumor suppressor gene after *TP53* [20]. PHTS is a genetic disease caused by *PTEN* mutations in the germline on chromosome 10 [21]. PHTS was recently codified in the ICD-10 coding system (Q85.81) in 2022 and condenses a group of disorders with similar clinical manifestations [22]. These disorders are named Cowden Syndrome, Proteus and Proteus-like Syndromes, and Bannayan–Riley–Ruvalcaba Syndrome [23,24].

Inheritance patterns seen for PHTS are autosomal dominant, but de novo mutations have been reported [25,26]. Up to 85% of adult PHTS patients reportedly have *PTEN* germline mutations, so some cannot be diagnosed based on molecular testing alone [27]. Patients with PHTS present with unique phenotypes, such as tissue overgrowth, macrocephaly, vascular anomalies, skin lesions, and multiple hamartomas (benign growths) [23,24]. Hamartomas are considered benign tumors that often cause no symptoms, but may be incidentally discovered due to an obstruction or dysfunction [28]. Interestingly, though hamartomas may not be cancerous themselves, they may represent a germline cancer predisposition for certain organ systems.

After PHTS was molecularly characterized in 1997, one subtype of PHTS, formerly referred to as Cowden Syndrome, was better defined by observable phenotypes such as macrocephaly, neuropsychological impairments such as autism spectrum disorder (ASD), and developmental delay [23,24,29]. In addition to ASD and neurodevelopmental conditions, PHTS studies have provided significant evidence that germline mutations in *PTEN* carry a much higher risk for several cancers when compared to the general population: breast (91%/13%), endometrial (48%/4.9%), thyroid (33%/1.5%), kidneys (30%/1.9%), colon (17%/5%), and skin (5%/2.1%) [25,26,30].

Since the literature on PTPRs is limited, and, to the best of our knowledge, there are no published reports on PTPRs with an incidental finding of PHTS, we were prompted to conduct a review of the most recent cases and report this first case connecting this unique intracranial brain tumor entity and genetic tumor syndrome.

## 3. Materials and Methods

### 3.1. Patient Sample Acquisition, Imaging, and Characterization

The patient consented to our accessing of their electronic health records for the analysis and display of data. The patient has consented to the publication of this data. Multiplanar, multisequence magnetic resonance imaging of the brain was performed before and after the administration of intravenous gadolinium-based contrast at the Huntsman Cancer Institute. Immunohistochemical staining was performed by ARUP. Tumor tissue was fixed in 10% neutral buffered formalin and paraffin embedded. Tissue was sectioned into 5 um thick sections and stained in their standard IHC staining services using the following LDT test numbers: 2003872, 2003493, and 2004127. DNA methylation was performed at the NYU Department of Molecular Pathology’s CLIA-certified laboratory, using the Illumina EPIC array and analyzed using the Heidelberg (DKFZ)-developed and NYU-clinically validated DNA methylation classifier in a CLIA-certified laboratory.

### 3.2. PTPR Literature Review

As a rare tumor with only a few hundred cases reported to date, our current understanding of PTPRs relies heavily on observational clinical case reports. PTPRs have only recently been given a distinct classification, much like other CNS tumors that continue to be defined and further classified through advancing technologies. As such, we performed a systematic literature review for PTPRs by searching the Web of Science and PubMed databases for reports published since 2020 using the term ‘papillary tumor pineal region’. Our research question focused on inconsistencies we observed in the diagnostic methods, treatments, and outcomes for PTPR patients. Our intent is to establish a future narrative for a meta-analysis.

**Figure 1:**
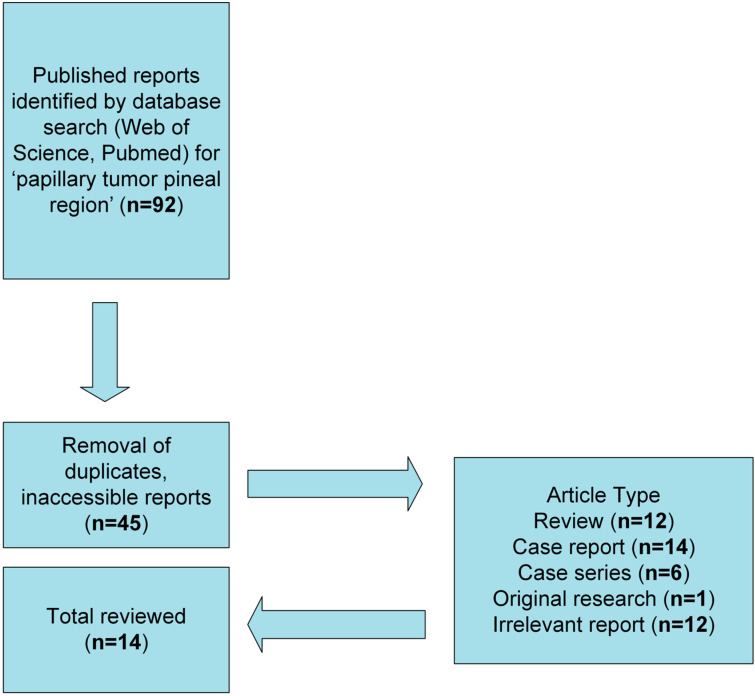
Flowchart as a visual diagram of the database search and strategy for the case study selection reviewed for “papillary tumor pineal region”.

Filtering cases reported from 2020–2024, we pulled articles that were duplicated in both databases and then condensed and further screened the articles for inclusion and exclusion criteria. The flow chart for the systematic search and retrieval process used for this review can be seen in Figure 1.

## 4. Results

### 4.1. Systematic Review of the Literatire Since 2020

A total of 45 articles were initially gleaned from the search after removing duplicates and inaccessible reports. These articles were then categorized as review articles, case reports, case series, original research, or irrelevant. This review aims to analyze only the recent case reports (n = 14), as this dominates the literature on PTPRs, and to avoid potential overlapping data from reviews and case series. To be included in the analysis using the listed findings from reviewed reports as seen in Table 1, at least one variable was required to be reported.

Demographics for our review show equal male and female distribution and ages ranging from 5–61. The mean age is 27.5 years and 100% reported hydrocephalus symptoms. Diagnostic methods reported for PTPRs appear to be widely variable, lacking consensus for characteristics beyond imaging and histological appearance and immunohistochemical staining, which overlap closely with other primary CNS tumors, such as ependymomas. The diagnostic data at large elucidates inconsistencies speculated for this rare tumor but has unexpectedly demonstrated limited molecular testing during the height of the molecular era, with only 3 out of 14 cases utilizing molecular methods to achieve a definitive diagnosis, see Table 2. The methods mentioned, such as chromosomal microarray, NGS, and WGMP, were indeed capable of distinguishing the PTPR tumors. Since PTPRs have shown potential for misdiagnoses at the scope alone, including 2 out of the 14 cases we reviewed, access to and use of these molecular tools is imperative for a definitive diagnosis for pineal region tumors.

Treatments for PTPR patients in these reports vary from surgical interventions and radiation to experimental chemotherapy. Since PTPRs cause obstructions that lead to secondary hydrocephalus, as seen in all the cases we reviewed, surgical interventions are required, including the use of shunts. However, the diversion technique known as endoscopic third ventriculostomy (ETV) as opposed to a shunt was more commonly utilized in the review reports (8/14), see Table 2.

Outcomes observed for PTPR cases reviewed are variable, and poor outcomes may be consistent with delayed intervention or unsuccessful treatments, see Table 2. Deaths reported could be more specifically related to delays in diagnosis, access to neurosurgical care or the use of unnecessary/improper treatments, but the case counts are too low to definitively determine the circumstances contributing to the outcomes.

### 4.2. Detailed Case Report

A 21-year-old male with macrocephaly presented to a critical access hospital with nausea and vomiting after 2 weeks of extreme headaches and vision disruption. He claimed to experience “pressure headaches” most of his life. Imaging at a rural emergency room revealed a lesion likely causing secondary obstructive hydrocephalus at the cerebral aqueduct in the third ventricle near the pineal region. The patient was transferred to a neurosurgery center. Pertinent childhood history included developmental delays, macrocephaly, and superficial lipomas. A pre-operative MRI scan showed enlarged lateral ventricles and revealed a rounded enhancing mass lesion within the floor of the third ventricle adjacent to the cerebral aqueduct, which measured 1.1 × 1.1 cm and required excision. There was no significant associated gradient susceptibility. Other non-mass like foci of enhancement were also revealed in early images and later identified as vascular anomalies.

After the initial biopsy, ETV, and a second surgery claiming GTR of the mass, histology revealed an initial diagnosis of ependymoma, WHO grade 3. Tumor markers (beta human chorionic gonadotropin, alpha fetoprotein, and alkaline phosphatase isoenzymes in both cerebral spinal fluid and serum) were within normal range. Cytology and imaging revealed no dissemination in the spine.

**Figure 2:**
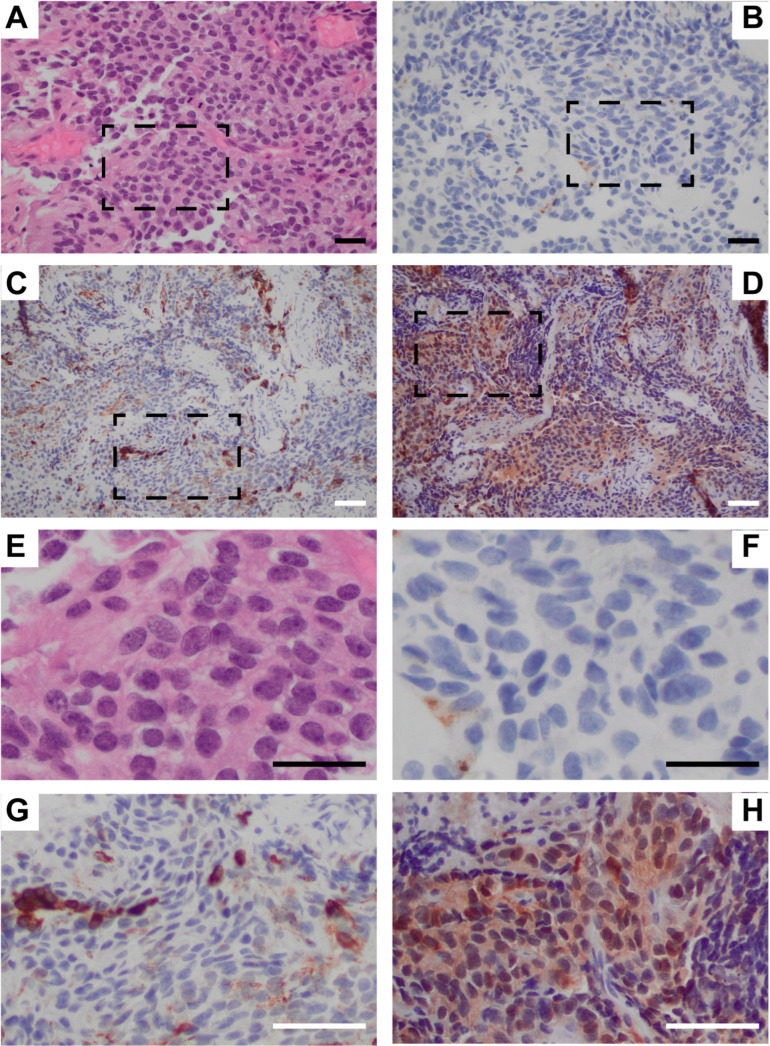
Histology and IHC used to identify tumor characteristics. (**A**) 40× initial H&E showing hypercellularity and mitotic activity, (**B**) 40× EMA, aids in distinguishing meningiomas, (**C**) 20× CAM 5.2, aids in distinguishing neuroendocrine carcinomas, (**D**) 20× S100, aids in identifying a tumor consisting of glial cells, (**E**) Dotted box from part A, (**F**) Dotted box from part B, (**G**) Dotted box from part C, (**H**) Dotted box from part D. Black scale bars = 50 μm, white scale bars = 100 μm.

The tumor tissue was grossly described as soft and tannish pink in color. Sections revealed a glial tumor of an ependymal lineage. The tumor was moderately hypercellular and contained oval nuclei with mild pleomorphism (Figure 2A,E). Perivascular pseudo rosettes and tubules were observed. There was elevated mitotic activity (3 mitoses/10 high powered field) and microvascular proliferation and necrosis were absent.

IHC results showed GFAP (Glial Fibrillary Acidic Protein): Faintly positive in some tumor cells, EMA (Epithelial Membrane Antigen): Positive for dot-like luminal cytoplasmic staining (Figure 2B,F), CAM5.2 (Calmodulin5.2): Positive in some tumor cells (mostly around vessels) (Figure 2C,G), Synaptophysin: Negative in tumor cells, S100: Weakly positive in some tumor cells (Figure 2D,H). Most of the tumor cell nuclei had retained H3K27me3.

**Figure 3:**
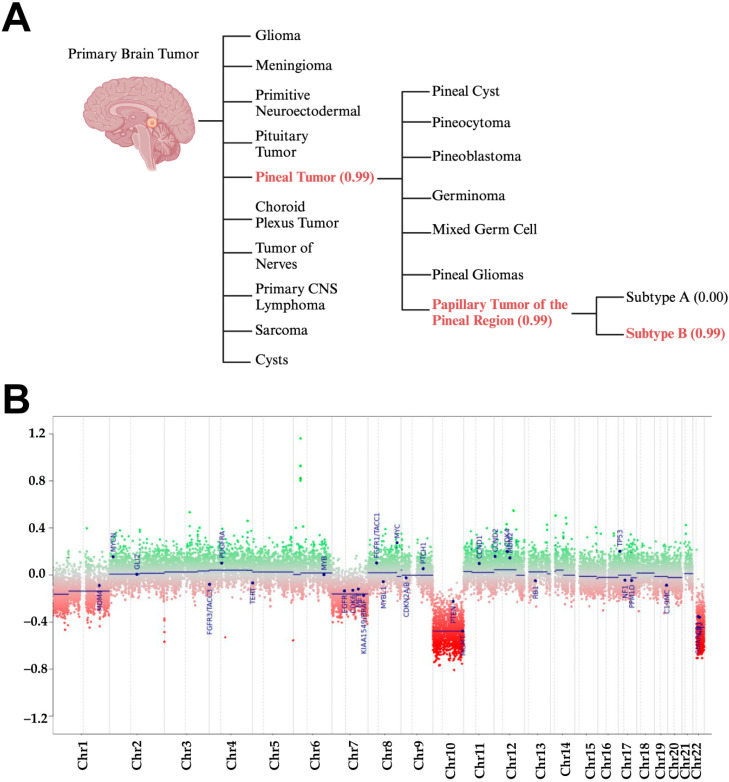
Whole-genome DNA methylation analysis revealed a distinct methylation class from other primary brain tumors and identified the proper subtype of PTPRs. (**A**) Methylation class and subclass based on global methylation patterns in the tumor. The numbers in parentheses indicate the calibrated methylation score. Any score ≥ 0.9 is considered a match. Throughout the decision tree process for the differential diagnosis, even the subtype B was identified with a high calibrated score of 0.99, (**B**) the copy number variation profile from the same methylation assay was generated with chromosomal regions either being amplified or lost in the tumor, 29 brain tumor relevant gene regions are highlighted for easier assessment, 0.0 indicates the baseline or diploid state.

Further diagnostic investigation was performed on the tumor tissue using a clinically validated WGMP using the Illumina EPIC BeadChip array (NYU Langone, Whole-Genome DNA Methylation Analysis). Using version 12.8 of the brain classifier algorithm, methylation patterns were able to provide a distinct methylation class (Figure 3A). The methylation patterns revealed the unique entity in the final diagnosis that was corrected after discharge; PTPR, subgroup B (calibrated score 0.99) (Figure 3A).

As part of the methylation assay, a copy number variation profile was generated to depict the relative loss and gain of chromosomes compared to a baseline diploid state. The most notable chromosomal losses in this tumor were in chromosomes 10 and 22. A total of 29 relevant brain tumor genes are indicated on the graph and notably PTEN and MGMT are located on chromosome 10 whereas SMARCB1 and NF2 are located on chromosome 22 (Figure 3B). These are all prominent tumor suppressor genes. Chromosomal 10 loss is often present in PTPRs.

Oncologists supplied a no-cost kit for additional genetic testing at the patient’s request for further evaluation. The laboratory sent the tumor specimen, and the patient supplied a normal-matched sample (saliva) that confirmed the pathogenic *PTEN* G132D missense mutation as a germline variant via NGS (Tempus/GeneDX, xT Solid Tumor + Normal Match 648 gene panel & xG+ 77 gene panel for confirmation). The oncologist noted that the patient exhibited phenotypes consistent with major and minor criteria for the Cowden Syndrome subtype of PHTS and referred him to genetic counseling.

The patient in this case was recommended by the tumor board for proton radiotherapy proceeding surgery. Due to a lack of symptoms, side effects, and potential negative outcomes, the patient opted out of this experimental therapy and requested more molecular testing that ultimately revealed his genetic condition. He had one genetic counseling appointment, and no other family members have been screened for similar mutations. Currently, he has mild to moderate headaches and is clinically stable at 28 months post-operation. The patient opted to remain under surveillance by the second neurosurgeon for the residual tumor. His surveillance includes bi-annual cranial MRI scans and annual spinal MRI scans with prospective plans for surgical intervention if/when the tumor grows large enough to be removed.

## 5. Discussion

This first case report of a PTPR case featuring PHTS combined with our systematic review offers clues into the associations behind both conditions. Since only one brain tumor is considered pathognomonic for PHTS, a benign dysplastic tumor of the cerebellum known to be a hamartoma called Lhermitte–Duclos disease [44], it begs the question: could one of the subgroups of PTPRs be more characteristic of a hamartoma forming early in development? With recent reports revealing a potential further delineation of the PTPR group, it is possible that aggression may be miscalculated.

This paper assists with laying the foundation for consensus over the molecular characterization of PTPRs in diagnostics, and for a conservative approach regarding unproven therapies. Surgical approaches to PTPR deserve further refinement as well, seeing as nearly 29% of patients undergo >1 surgery related to PTPR symptoms and conditions. In addition, with reflection on our case report, knowing that PHTS cancer risks are extraordinary, exhausting available therapies ahead of future disease progression would be doing a substantial disservice to the patient.

The molecular characterization of tumors is becoming more commonplace, yet it begs the question of whether the cost of germline next generation sequencing is justified. The costs for targeted gene panel sequencing with its indirect costs of analysis and counseling are affordable even without insurance coverage and should continue to decrease in the future. The benefits of sequencing could guide personalized treatment plans. In the highlighted case of the PTPR with a germline *PTEN* pathogenic variant, there are several FDA-approved drugs that target the activated PI3 kinase pathway that could be used. Expanded sequencing to include the entire exome or genome could reveal neoantigens that can be targeted with novel immunotherapies. While there is insufficient data to demonstrate the long-term benefits and patient outcomes as a result of incorporated genomic sequencing in patients with PTPR, we do know that the immediate impact of identifying a *PTEN* germline pathogenic variant has resulted in genetic testing in immediate family members and an awareness and vigilance to screen sooner and more frequently for cancer. Early detection in any cancer has a significant cost–benefit ratio. Treatment costs for stage 1 cancers are typically a tenth of the cost to treat stage 4 cancers. Survival rates triple for stage 1 cancers compared to stage 4 cancers.

An important aspect of the case report to mention is that the tumor was initially considered a GTR, but a thorough review of images 18 months post-operation identified the very small residual mass. As such, it is imperative to state that neurosurgeons must order MRI images in 1–2 mm slice thicknesses for very small tumor resections following surgery to best confirm GTR.

## 6. Conclusions

PTPR incidence remains low, offering challenges in research, diagnostic methods, and treatments. Inconsistencies remain in diagnostic methods and treatments, contributing to the wide variability seen in patient outcomes. Future research depends on clinicians utilizing molecular diagnostic approaches in a personalized fashion and making further contributions by submitting case studies confirmed for PTPR to assist researchers with measuring patient outcomes.

## Figures and Tables

**Table 1:** Single case reports of PTPR and selected demographics and variables.

Author, Year	Age	M/F	HCP	DX	TX	Outcome(s)
Kennedy et al., 2023 [31]	61	F	Yes	Imaging, histology, IHC, chromosomal microarray	Surgery×2 (STR, GTR), shunt	Residual/ recurrence; GTR, Stable 1 year post-op after 2nd surgery
* Mehta et al., 2021 [32]	29	M	Yes	Imaging, histology, IHC	Surgery×2 (STR), shunt	Tumor progression seen 6 months post-op after 2nd surgery
Nemir et al., 2022 [33]	9	F	Yes	Imaging, histology, IHC, tumor markers	Surgery (GTR), ETV, PRT	Stable, no recurrences 78 months post-op
Gutpa et al., 2021 [34]	5	M	Yes	Imaging, histology, IHC	Surgery (GTR)	Not reported
Damgacı et al., 2020 [35]	17	F	Yes	Imaging, histology	Surgery (GTR)	Not reported
Bechri et al., 2021 [36]	26	F	Yes	Imaging, histology, IHC, tumor markers, cytology	SRS, ETV	Stable, 60% reduction in tumor volume 1 year post-op
* Assi et al., 2021 [8]	25	M	Yes	Imaging, histology, IHC, next generation sequencing	Surgery×3; 1st GTR, RT, everolimus, dexamethasone 2nd ETV 3rd GTR	Multiple brain masses in cerebellum and pineal region excised, 43 months stable on everolimus
Schwartz et al., 2025 [37]	52	F	Yes	Imaging, histology, DNA methylation	Surgery, ETV	Not reported
Marfia et al., 2020 [38]	40	M	Yes	Imaging, histology, IHC	Surgery, ETV, RT	Stable, reduced tumor size 20 months post-op
Dhandapani et al., 2024 [39]	16	F	Yes	Imaging, histology	Surgery (GTR), ETV, RT	Stable, no neurological deficits
Bromfield et al., 2020 [40]	24	M	Yes	Imaging, histology, IHC	Surgery (STR), ETV	Death at 3 months post-op, no post mortem exam
Gupta et al., 2020 [41]	25	M	Yes	Imaging, histology, IHC	Surgery	Death at 4 days post-op via cardiac arrest
Anvari et al., 2024 [42]	46	M	Yes	Imaging, histology, IHC	Shunt, surgery×2 (STR), vincristine, carboplatin,	1st surgery; stable 14 months post-RT, 2nd surgery; disseminated disease, stable 6 months post-op after chemotherapy
Mathkour et al., 2021 [43]	10	F	Yes	Imaging, histology, IHC, cytology	Shunt after ETV failed, surgery (GTR)	Stable, no recurrences at 3 years post-op

Hydrocephalus (HCP), immunohistochemistry (IHC), subtotal resection (STR), gross total resection (GTR), endoscopic third ventriculostomy (ETV), radiotherapy (RT), proton radiotherapy (PRT), stereotactic radiosurgery (SRS). * indicates case studies that were initially misdiagnosed.

**Table 2:** Study parameters.

Variable Analyzed	Values
Age	Mean: 27.5 years
Gender (M/F)	Ratio: 50/50
Hydrocephalus	100%
DX	Imaging: 100% Histology: 100% IHC: (3) 21.42% Cytology: (2) 14.29% Tumor markers: (2) 14.29% Molecular: (3) 21.42%
TX	Surgery: 93% >1 surgery: 28.57% Shunt: 28.57% ETV: 57.14% RT: 35.71% Chemotherapy/kinase inhibitor: 14.29%
Outcomes	Stable: 57.14% Ongoing/progressive disease: 35.71% Death: 14.29% Not reported: 21.42%

## Data Availability

The datasets presented in this article are not readily available because the data remain part of an ongoing study. Requests to access the datasets should be directed to N.O.
